# The *Times* on the Insanity of George III

**Published:** 1863-10

**Authors:** 


### THE "*TIMES*" ON THE INSANITY OF GEORGE III.

The following valuable, comprehensive, and peculiarly interesting observations on the lunacy of George III., are from a review in the *Times* (14th August) of Mr. Phillimore's History of England. They are unrivalled in the apt appreciation of the influence which the lunacy of the King exercised upon the affairs of the nation:—

"The character of George III. we are not going to defend. We do not think that he was a great man or a great King, or much better than a sturdy British farmer of the uneducated type. He was narrow in his views, very cunning, fond of power, and intolerant of opposition, even to vengeance. The mode in which at the beginning of his reign he threw himself into the arms of Bute showed a singular want of understanding. Bute knew nothing of statesmanship, and he was known only as a handsome voluptuary. His apparent position was that of Groom of the Stole to the young Prince who was about to ascend the throne, as he had been Lord of the Bedchamber to the young Prince's father, Frederick; his real position was that of paramour to George's mother, the Princess of Wales. Soon after the decease of the Prince of Wales he gained upon the favour of the Princess, and had supreme power over her son. It was for this man, because the Princess, his mother, had a passion for him, that George III., when he ascended the throne, discarded such a statesman as Pitt, whose policy was just then crowning the nation with glory. One can have no great faith in the judgment of a King who could thus inaugurate his reign by the triumph of a personal friendship over superb statesmanship; and so far as Mr. Phillimore in the present volume is engaged in showing the folly of such conduct and the evils to which it led, we have no substantial difference with him. We object, however, to the mode in which, as he relates event after event, he heaps contumely on the head of poor George III. He was mad, and the historians forget this. They treat him as if in his lucid intervals he were altogether a sane man. This is the perfection of commonplace. This is that legal bondage and chicanery of detail which Mr. Phillimore pretends to dislike. Legally, a man who is only a little mad, or only occasionally mad, is held to be responsible, and is allowed, for example, to manage his property. But it is the province of history to give not merely legal judgments—the historian has to pronounce moral judgments. In that case what can be said of George III.? Can we draw a sharp line and say here he was mad, there he had no taint of madness in him? Not so; the life of the madman, in so far as it is comprehensible to the moralist, must be regarded as all more or less infected. In George III. there was the taint from first to last. His eternal cunning, for example—was it not essentially the small cunning of a madman? Historians treat of this Royal insanity with the most astonishing coolness, as if it were an ordinary event, that no more disturbed or could disturb the State than a fit of sneezing or of the gout. The machine of Government went on like a clock, and the historians, therefore, write as if nothing very extraordinary took place. But where else save in England could the King become mad, and yet no effect be observed in the action of the Legislature? In other States he would have been deposed, or some means would have been taken to put him out of the way. In England they simply appoint a Regent, and wait for his recovery. The madness of a King upon the throne of England goes for little or nothing in the histories of the period. To our minds it produces in history a situation as tragically interesting as the contemporary madness of a whole people across the Channel. In 1789 the French people broke mad for a time, and their madness exhausted itself in the Reign of Terror and the wars of the Republic. In 1788, not the English people, but the English King broke mad, and such is the nature of the British Constitution, that his madness gave to us a reign of dulness. One can trace distinctly how the Royal mania affected the policy of our wisest Ministers. We cannot do this, and we cannot do that, they argued, for fear of exciting the King. The King was mad against Fox, the King was mad against Catholic Relief. But historians treat of this madness as if it were only an exaggerated form of ordinary antipathy. It was much more; it was a veritable insanity. And we maintain that this insanity, which after 1788 was palpable, and had a visible influence on the conduct of Ministers, before 1788 was latent, may be traced in the King's conduct, and had its effect on the Court. In this view it is absurd to indulge in bitter remarks on the King's conduct. Who can be bitter against a man visited with so great an affliction? His affliction, it is true, was the nation's affliction; but are we on that account to turn upon him and rend him in pieces? Surely, we may rather dwell upon his good qualities, which were strikingly exemplified, and which, while he lived, secured the love and reverence of the great body of his people. His homely virtues, his temperance, his unaffected piety, are not to be forgotten. It was, indeed, an excess of this homely virtue that made him begin his reign with a mistake. The affectionate young fellow was perfectly submissive to his mother; and was too dull to take a path for himself. This dulness was part of his malady, and should excite commiseration rather than scorn. Unfortunately people do not believe in madness unless it shows itself in outrageous conduct. If George III. had exhibited the vices of his eldest son, everybody would be willing to treat of his life as one long lunacy. But because he was sober, virtuous, pious, steady-going, and his madness showed itself in stupidity, it is not understood to be madness except when the fits came on. So it happens that men who are only stupid in their cups get the credit of not being at all drunk."

